# Supplementary material for: Phosphatidylinositol-4-phosphate controls autophagosome formation in Arabidopsis thaliana
Source: Nat Commun. 2022 Jul 28;13:4385. doi: 10.1038/s41467-022-32109-2 (PMC9334301; doi:10.1038/s41467-022-32109-2)
Supplement: Supplementary file 1 — Supplementary Information [file 41467_2022_32109_MOESM1_ESM.pdf]

Phosphatidylinositol-4-phosphate controls autophagosome formation in *Arabidopsis thaliana*

Supplementary file

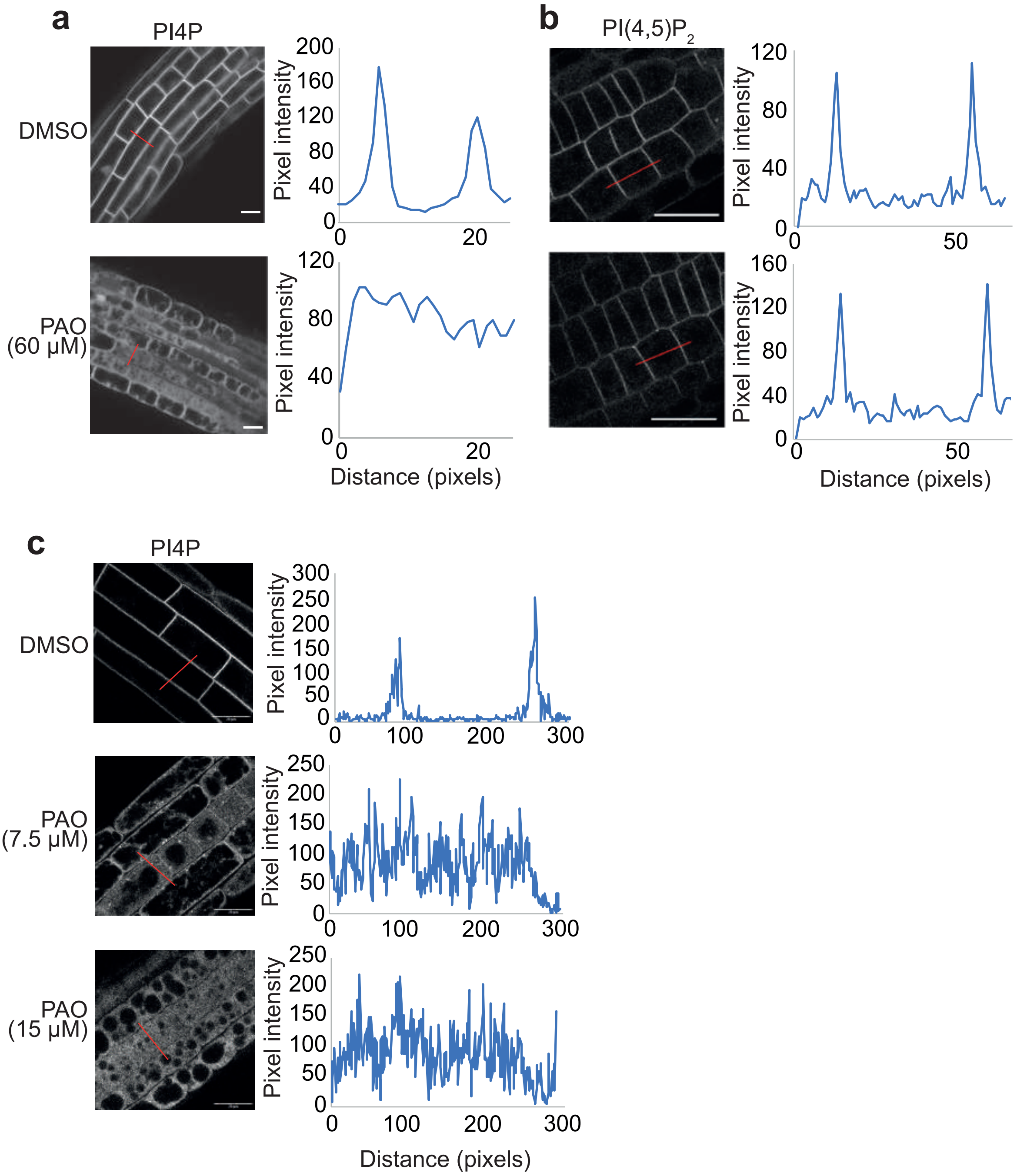

Supplementary Figure 1. **PAO treatment affects PI4P synthesis, without affecting that of the PI4,5P$_2$.** Seedlings were grown for 7 days in nutrient rich MS plates and then transferred to nutrient deprived (-NC, darkness) medium to induce autophagy. Confocal microscopy analyses show that PAO causes a mislocalization of a PI4P biosensor from the PM to the cytosol indicating a sharp reduction in the pool of PI4P at this compartment. In contrast, PAO has no effect on the pool of PI4,5P$_2$ in these conditions. **a, b** Confocal images of a PI4P biosensor (1xPH^{OSBP}, **a**) or a PI(4,5)P$_2$ biosensor (2xPH^{PLC}, **b**) treated for 30 minutes with PAO 60 µM compared to DMSO as control. **c** Confocal images of a PI4P biosensor (1xPH^{OSBP}) treated for 3 hours with PAO at 7,5 or 15 µM compared to DMSO as control. Graphics represent the localization of the probes by ploting its signal intensity within the drawn area (red line). Results present representative images of n=3 independent experiments. Scale bar, 20 µm.

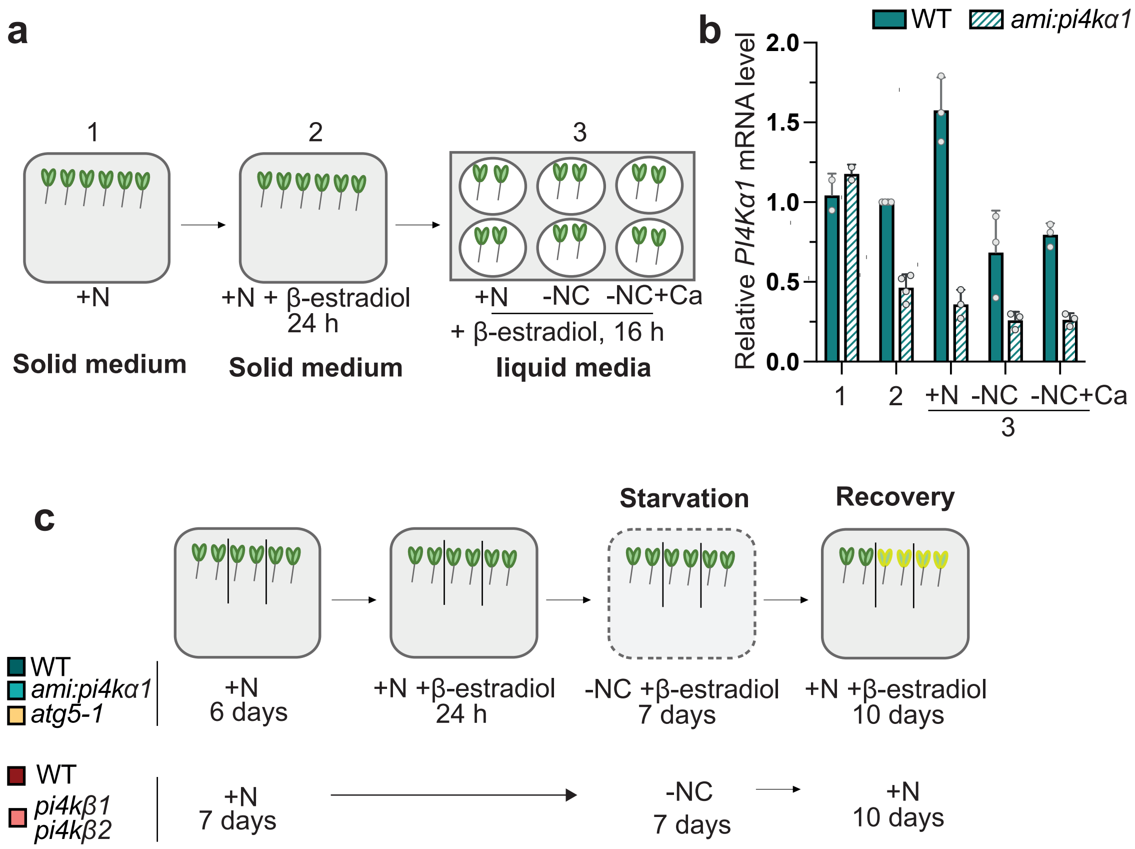

Supplementary Figure 2. **Characterization of the *amiRNA:PI4Kα1* line and experimental procedures of nutrient deprivation recovery experiments. a, b** *amiRNA:PI4Kα1* lines show a reduced *PI4Kα1* expression compared to WT plants. Schematics of the experimental procedures (**a**). Plants were grown on MS agar plates for 6 days (**a**), then transferred to MS plates supplemented with 10 µM β-estradiol for 24h to induce amiRNA expression (**b**). Seedlings were then transferred to different liquid media for 16 hours, all supplemented with 10 µM β-estradiol: nutrient rich (+N), nutrient starvation (-NC), or nutrient starvation medium supplemented with 1 µM concanamycin A (-NC+Ca). Samples were collected in each conditions and RNA were extracted and analyzed by RT-qPCR (**b**). The expression of *PI4Kα1* relative to WT in condition **2** (set to 1 in each experiments), was compared between the *amiRNA:PI4Kα1* line and WT plants in all conditions. Results are presented as the average of mRNA level with SD (number of independent experiments: n=2 for condition **1**, n=4 for conditions **2**, n=3 for conditions **3**). **c** Schematics of the experimental procedures followed in **Figure 2a**. Col-0 (WT), *amiRNA:PI4Kα1* and *atg5-1* were grown on MS agar plates for 6 days, then transferred to MS agar plates supplemented with 10 µM β-estradiol for 24 hours. Seedlings were then transferred to nutrient deprived (-NC) MS agar plates in the obscurity supplemented with 10 µM β-estradiol for 7 days to induce autophagy and then transferred back to full MS medium agar plates supplemented with 10 µM β-estradiol and in normal light conditions for 10 days for recovery. In the other set of samples, 7-day-old Col-0 (WT) and *pi4kβ1 pi4kβ2* double mutant plants were directly transferred from full MS plates to nutrient deprived (-NC) MS agar plates in the obscurity for 7 days to induce autophagy, and then transferred back to full MS medium agar plates in normal light conditions for 10 days for recovery.

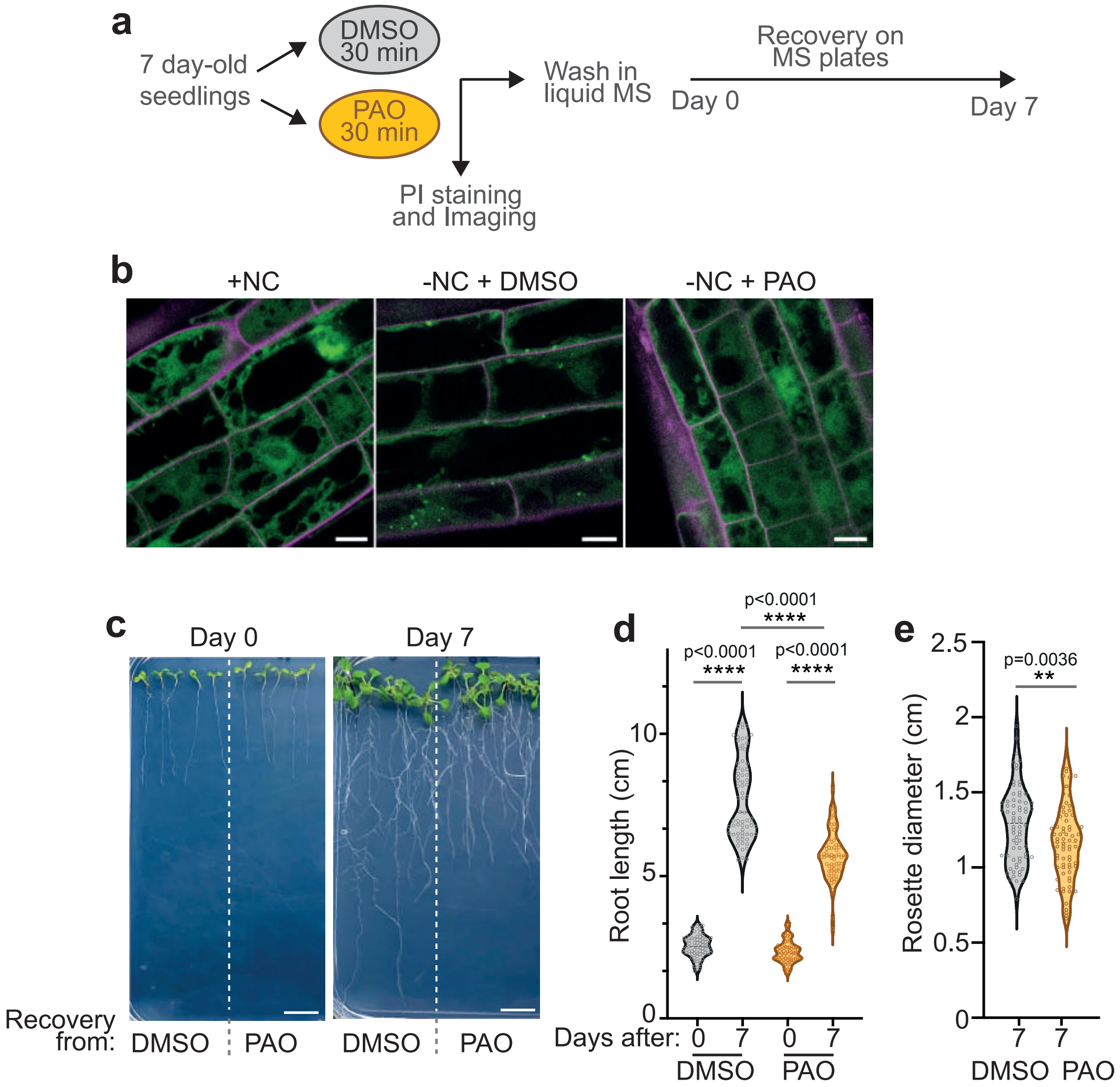

Supplementary Figure 3. **PAO treatment does not lead to cell death. a** Schematic of the experimental procedure for testing the effect of PAO on plant physiology. 7-day-old seedlings of the GFP-ATG8a line were transfered for 30 minutes to nutrient-deprived liquid medium containing 60 µM PAO or DMSO as control. Immediately upon treatment a subset of plants were stained with propidium iodide (PI) and imaged by confocal microscopy as described in (**b**). For the remaining plants, treatments were washed off by placing plants to +N liquid medium and plants were then transfered to +N MS plates for recovery during 7 days. **b** Representative confocal images of roots stained with PI in control conditions (+NC) or after 30 minutes in -NC liquid medium supplemented with either DMSO or PAO as described in (**a**). Snapshots of **Supplementary Movies 1-3**. In -NC + DMSO, autophagy is induced and GFP-ATG8a puncta are formed; the addition of PAO prevents the formation of puncta. In all conditions, PI staining was observed in the apoplast indicating cell integrity (in dead cells, PI enters the cells and stains the cytosol and nuclei). PI stainig was performed on a total of n=7 (+NC), n=7 (-NC+DMSO) or n=8 (-NC+PAO) independent plants over 2 independent experiments. Scale bars: 10 µm. **c-e** Seedlings treated with PAO show no sign of death after 7 days of recovery post treatment. Although root length and rosette size are reduced in PAO-treated plants than in control plants after 7 days of recovery, PAO-treated seedlings recovered and resumed root and rosette growth compared to Day 0. These results show that PAO treatment did not compromise cell viability. Scale bars, 1 cm. **d, e** Quantification of (**c**). Results represent Violin plots with individual values of n=70 biological replicates with data collected over 2 independent experiments. Statistical differences between conditions were assessed using two-tailed unpaired t-test, exact p values are provided in the source data file.

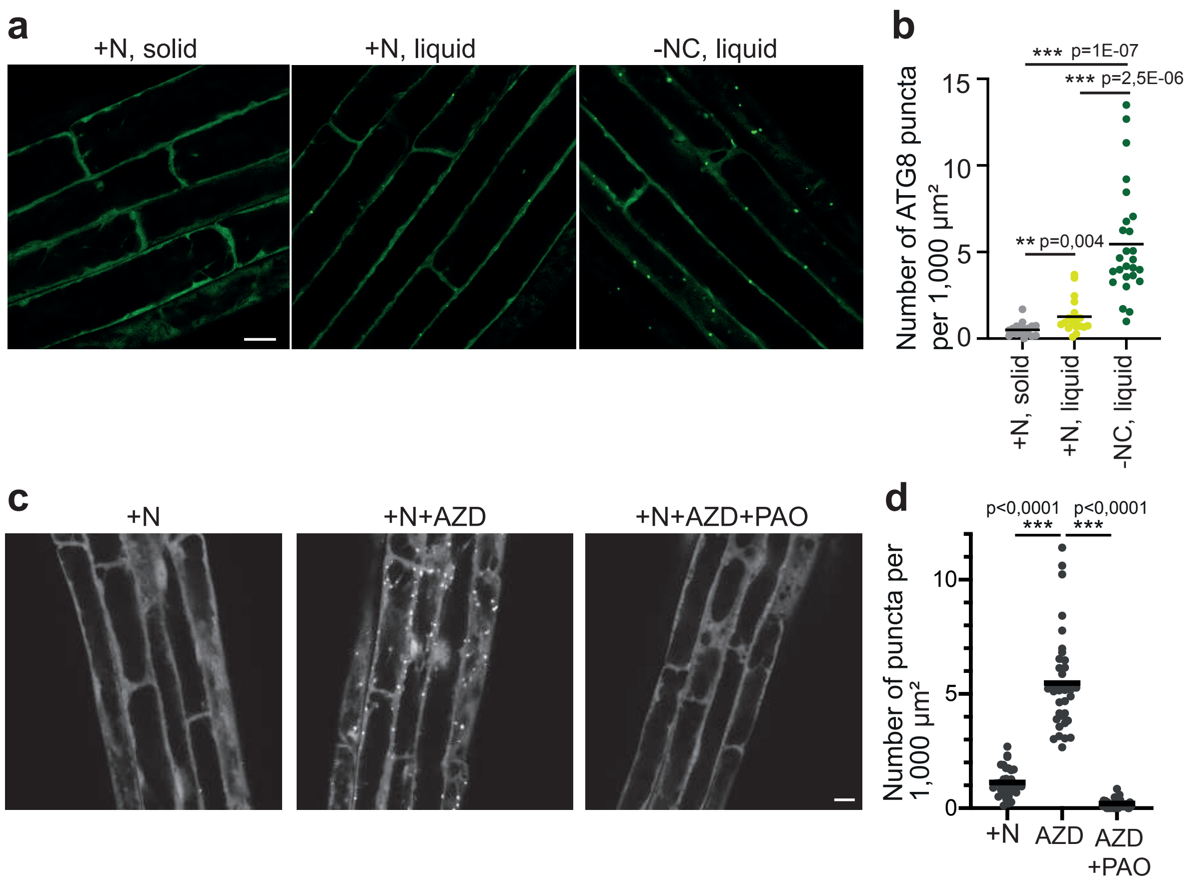

Supplementary Figure 4. **Effects of plant transfer, nutrient starvation and AZD on the number of GFP-ATG8a puncta. a, b** The number of GFP-ATG8a puncta significantly increases in -NC liquid conditions compared to +N solid or +N liquid conditions. **a** Confocal images of roots from Arabidopsis plants expressing GFP-ATG8a. Images of 7-day-old seedlings grown in nutrient rich MS plates (+N, solid); after 30 minutes in rich liquid medium (+N, liquid) or after 30 minutes in nutrient-deprived liquid medim in darkness (-NC, liquid). Scale bar: 10 µm. **b** Quantification of autophagic structures in conditions presented in (**c**). Results are presented as number of puncta per 1,000 µm² of root area. For +N solid, n=18 images were examined over 5 independent biological replicates; for +N liquid, n=19 images were examined over 5 independent biological replicates; for -NC liquid, n=26 images were examined over 6 independent biological replicates. Results present all values, bar is mean. Statistical differences between conditions were assessed using unpaired two-tailed t-test. **c-d Inhibition of PI4K activity blocks TOR-induced autophagosome formation. c** Confocal images of 7-day-old 35S::GFP-ATG8a seedlings treated for 30 min in liquid nutrient rich (+N) medium compared to a liquid rich medium supplemented with 1 µM AZD (TOR inhibitor) in control conditions (+N+AZD) or with PAO (+N+AZD+PAO 60 µM). Scale bar, 10 µm. **d** Quantification of (**c**). Results are presented as number of puncta per 1,000 µm² of root area, n=37 distinct replicates examined over 3 independent experiments. Results present all values, bar is mean. One-way ANOVA analyses showed statistical differences among the 3 conditions (+N, AZD, AZD+PAO); two-tailed t-test with a Bonferroni correction was used as a post-hoc test showing statistical differences when +N or AZD+PAO conditions were compared to AZD, exact p values are provided in the source data file.

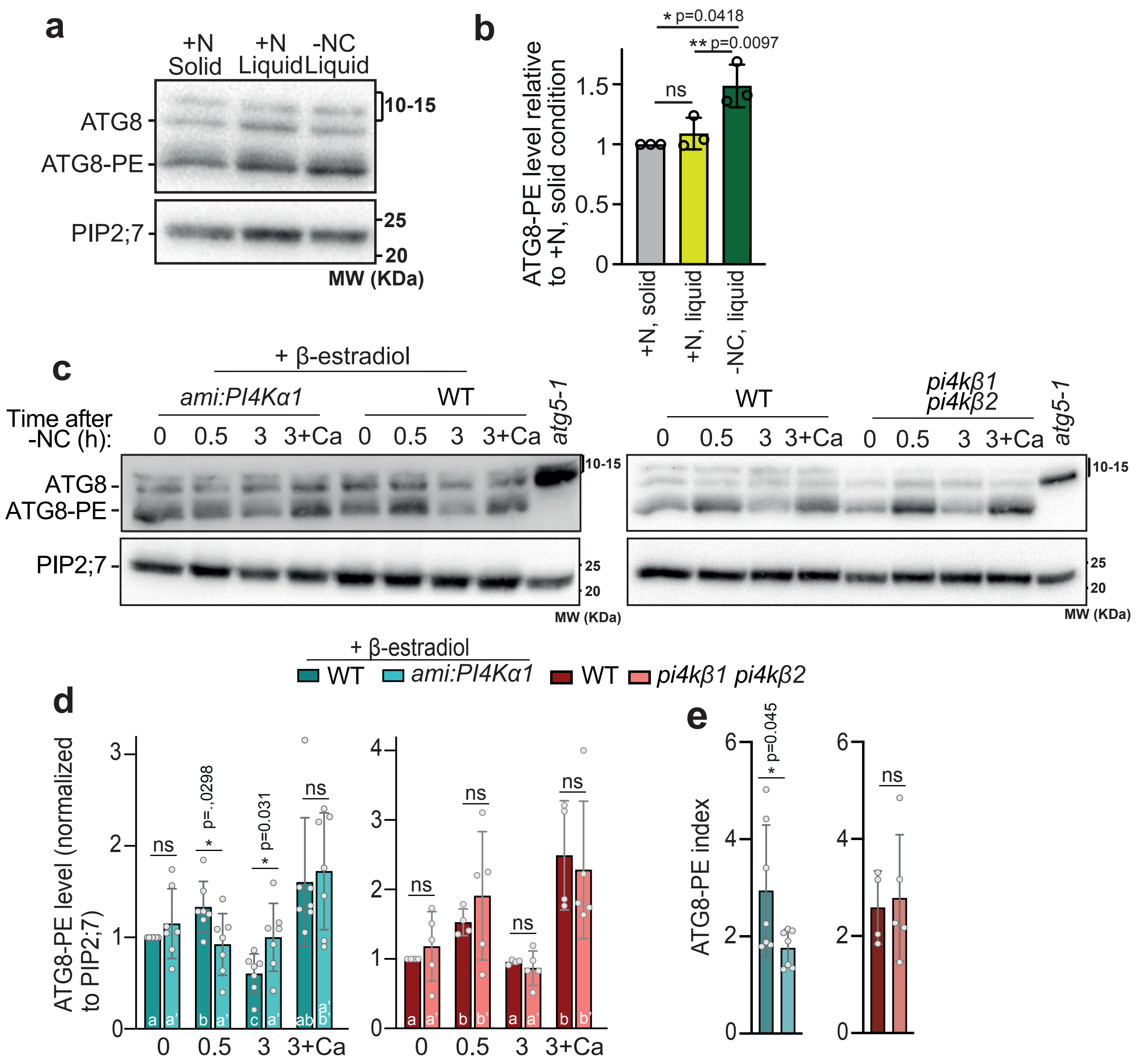

Supplementary Figure 5. **ATG8 lipidation is affected in the amiRNA:*PI4Kα1* mutant but not in the *pi4kβ* mutant. a,b** The level of ATG8-PE significantly increases in -NC liquid conditions compared to +N solid or +N liquid conditions. Immunoblot analyses of microsomes from roots of 8-day-old WT seedlings collected directly on MS plates (+N, solid) or after 0.5 h in either rich liquid medium (+N, liquid) or nutrient-depleted liquid medium in darkness (-NC, liquid). **a** Immunoblot analyses were performed using an anti-ATG8 antibody. PIP2;7 was used as loading control and normalization factor. Uncropped blots in Source Data. **b** Quantification of (**a**). Results present the average of the level of ATG8-PE band intensities normalized to that of PIP2;7 ± SD as well as individual values (n=3 independent biological experiments) with paired, two-tailed t-test. **c** ATG8 and ATG8-PE levels in the microsomal fraction of roots from WT plants compared to that of *amiRNA:PI4Kα1* or *pi4kβ1 pi4kβ2*. Immunoblot analyses were performed as described in (**a**). To compare the level of ATG8-PE between WT and the *amiRNA:PI4Kα1* line*,* 6-day-old seedlings were transferred to liquid +N medium supplemented with β-estradiol (10 µM) for 24 h. Then, plants were transferred to -NC liquid medium in darkness (with 10µM β-estradiol) for the indicated times in control conditions or supplemented with concanamycin A (1 µM, 3+Ca). To compare the level of ATG8-PE bewteen WT and the *pi4kβ1 pi4kβ2* line, 8-day-old seedlings grown on MS +N plates were either collected directly (0), or transferred to -NC liquid medium in conditions described above. Uncropped blots in Source Data. **d,e** Quantification of the ATG8-PE level in conditions presented in (**c**). **d** Results present the average of the level of ATG8-PE band intensities, normalized by the level of PIP2;7 and relative to the level of ATG8-PE in WT plants in rich conditions which was set to 1, ± SD (for WT vs. *amiRNA:PI4Kα1,* n=7 independent experiments ; for WT vs. *pi4kβ1 pi4kβ2,* n=4 independent experiments for WT, n=5 independent replicates in 4 independent experiments for *pi4kβ1 pi4kβ2*). Statistical analyses show significant differences bewteen data groups, indicated by different letters (a-c; a'-c'), (two-tailed paired t-test for comparison bewteen conditions in a given line; two-tailed unpaired t-test for comparison between the mutant and WT lines; ns, non significant). **e** The ATG8-PE index represents the ratio of ATG8-PE in -NC 3h+Ca conditions (3+Ca) divided by the level of ATG8-PE in -NC 3h (3) which reflects the net rate of ATG8-PE degraded after 3 hours of autophagy induction. Results present the average of ATG8-PE index ± SD as well as all independent values. Number of replicates and independent experiments are as in (**d**). Statistical differences were assessed using two-tailed unpaired t-test. ns, non significant.

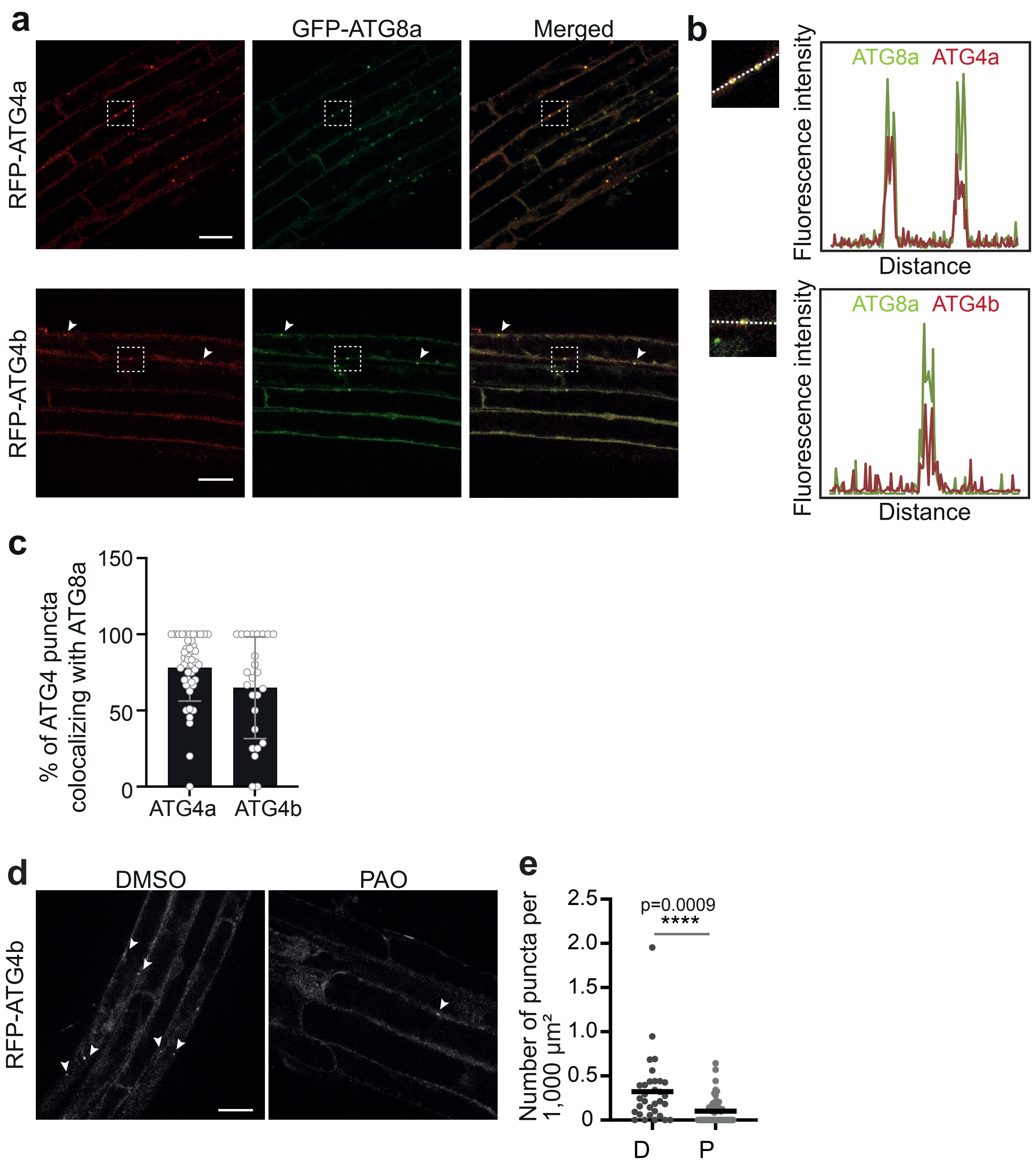

Supplementary Figure 6. **ATG4a and ATG4b isoforms are recruited to ATG8a-positive autophagic structures. a** Confocal images of Arabidopsis roots co-expressing GFP-ATG8a and RFP-ATG4a or RFP-ATG4b. 7-day-old seedlings were transferred to liquid nutrient depleted medium (-NC) for 30 minutes. Scale bar: 20 µm. **b** Graphics represent the signal intensity of the probes within the drawn area (dotted white line), created using the “plot profile” feature (Fiji software). **c** RFP-ATG4a and RFP-ATG4b colocalization in percentage of puncta with both RFP-ATG4 and GFP-ATG8 signal over total ATG4 puncta. Results represent the average with SD and all individual values; n=47 images for ATG4a and n=27 images for ATG4b, examined over 3 independent experiments. **d** RFP-ATG4b also localizes to dot like structures in control conditions but not in PI4K inhibition conditions. Confocal images if Arabidopsis roots expressing RFP-ATG4b. 7-day-old seedlings were transferred to liquid nutrient depleted medium (-NC) in control conditions (DMSO) or in PI4K inhibition conditions (PAO 60 µM) for 30 minutes. Scale bar: 20 µm. **e** Quantification of RFP-ATG4b dot-like structures in conditions presented in (**d**). Results as presented as number of puncta per per 1,000 µm² of root area, 3 independent experiments were performed, n=35 replicates (images) for DMSO (D) and n=52 replicates (images) for PAO (P). Statistical differences between conditions were assessed using two-tailed unpaired t-test.

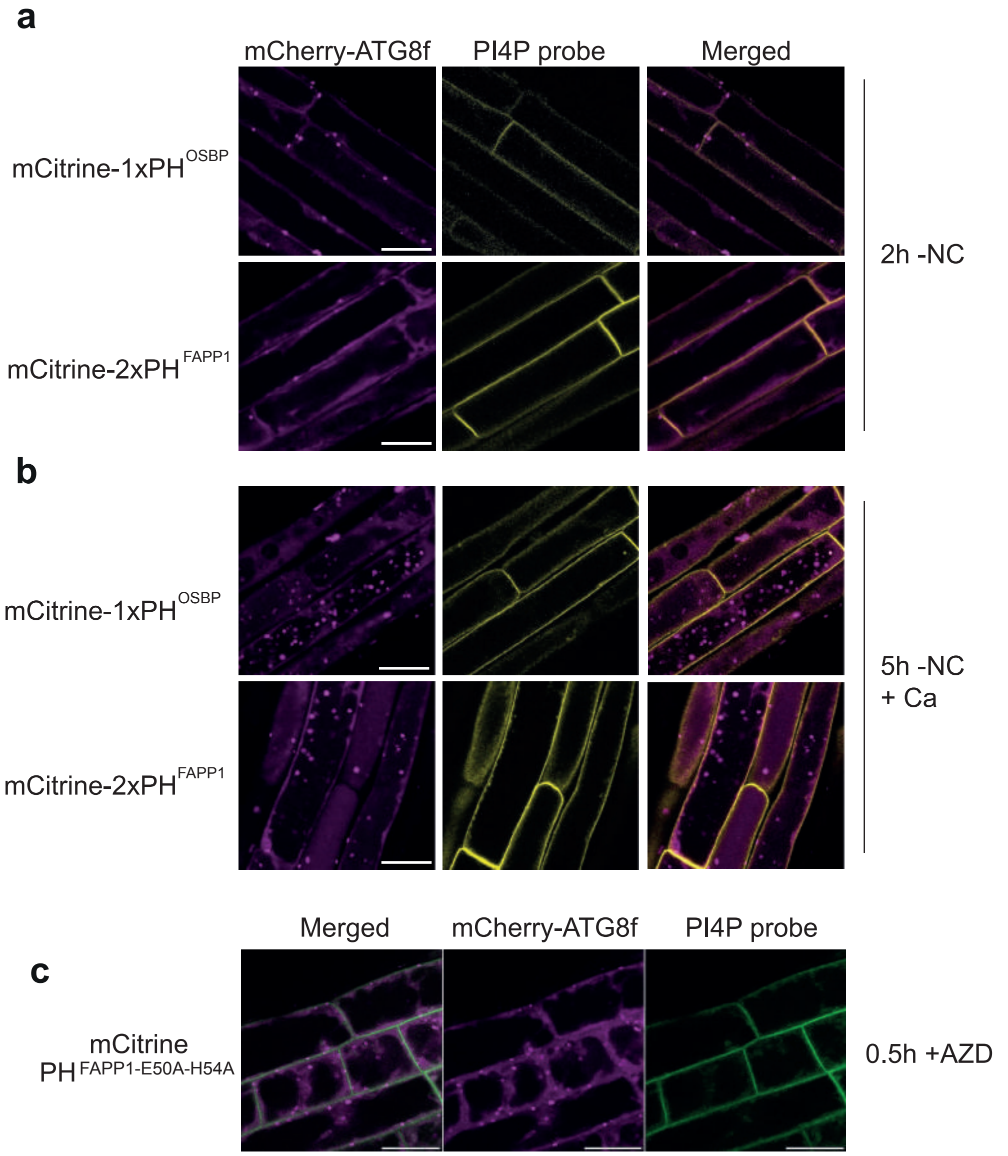

Supplementary Figure 7. **Co-localization analyses of autophagic structures and PI4P biosensors.** Representative confocal images of 7-day-old seedlings co-expressing mCherry-ATG8f and the PI4P-binding probes mCitrine-1xPH^{OSBP}, mCitrine-2xPH^{FAPP1} or mCitrine-PH$^{FAPP1\text{-}E50A\text{-}H54A}$. **a** Plants were placed in liquid MS medium deprived of nutrients (-NC) for 2 hours in control conditions. **b** Plants were placed in liquid MS medium deprived of nutrients (-NC) for 5 hours, supplemented with concanamycin A (Ca, 1 µM). **c** Plants were placed in liquid MS medium with 1 µM AZD for 0.5 hour. Scale bar: 20 µm. Images are representative of 2 independent experiments with: for mCitrine-1xPH^{OSBP}, n=8 (2h -NC) or n=11 (5h -NC + Ca) distinct replicates; for mCitrine-2xPH^{FAPP1}, n=14 (2h -NC) or n=18 (5h -NC + Ca) distinct replicates; for mCitrine-PH$^{FAPP1\text{-}E50A\text{-}H54A}$, n=20 distinct replicates.

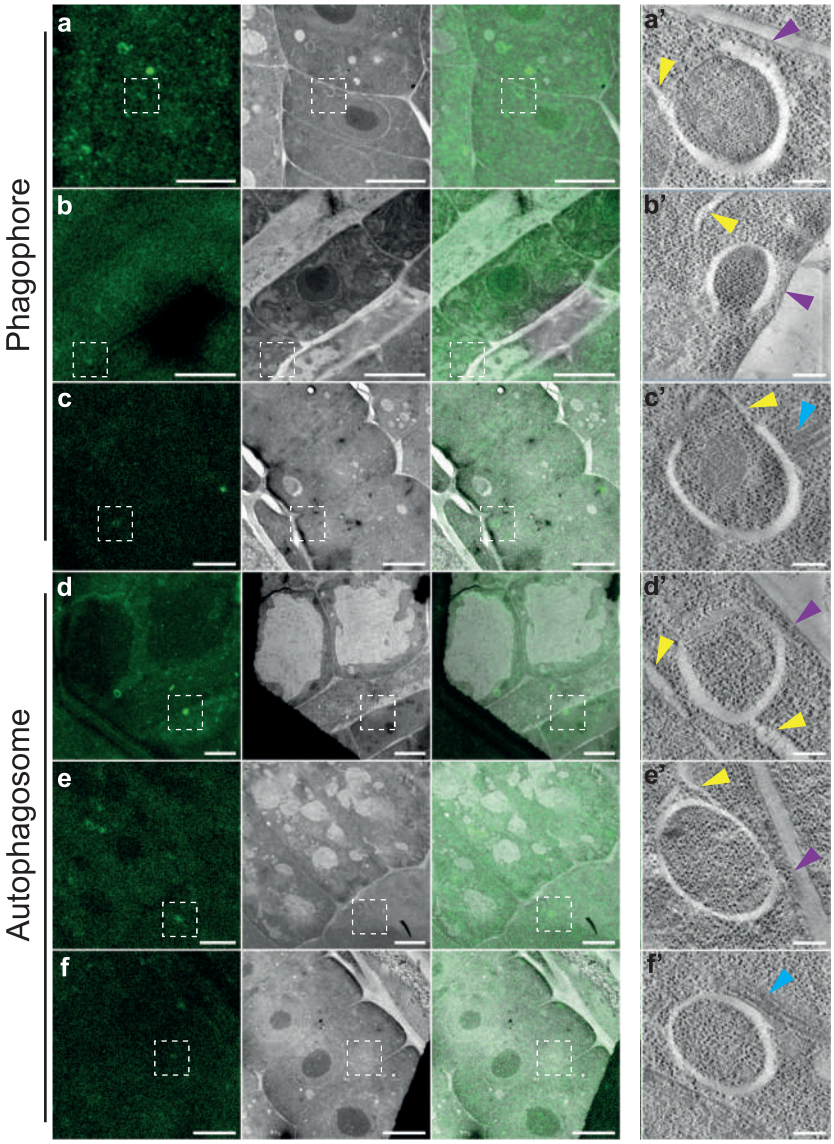

Supplementary Fig. 8. **CLEM analyses of the cellular environment of autophagy structures.** Confocal, transmission electron microscopy and correlated images of phagophores (**a**, **b**, **c**) and autophagosome (**d**, **e**, **f**) labeled with GFP-ATG8a. White dashed-squares indicate the autophagic structures selected for subsequent electron tomography (**a'-f'**).Tomograms reveal the proximity of autophagy structures to the ER (yellow arrowheads), the PM (purple arrows), or the Golgi Apparatus (blue arrowheads). See **figure 7** for additional information and quantification. Scale bars: (**a-f**) 5 µm; (**a'-f'**) 0,2 µm.

Supplementary Table 1: primers used in this study

Gene and usage	Primers (F/R)	Sequence
**ATG4a (AT2G44140)**		
Cloning Complete cds	F	GGGGACAGCTTTCTTGTACAAAGTGGCTATGAAGGCTTTATGTGATAGATTTGTTC
	R	GGGGACAACTTTGTATAATAAAGTTGTGATTGTTAAAATGTTTGACGATGATCATGA
**ATG4b (AT3G59950)**		
Cloning Complete cds	F	GGGGACAGCTTTCTTGTACAAAGTGGCTATGAAGGCTATATGTGATAGATTTGTTC
	R	GGGGACAACTTTGTATAATAAAGTTGTGAAAAGAATGGCTAGGAGAAGTGT
**PI4Ka (AT1G49340)**		
Cloning Inducible miRNA 1	I miR-s	gaTGATATTCGAACACACACCTGtctctcttttgtattcc
	II miR-a	gaCAGGTGTGTGTTCGAATATCAtcaaagagaatcaatga
	III miR*s	gaCAAGTGTGTGTTCCAATATCTtcacaggtcgtgatatg
	IV miR*a	gaAGATATTGGAACACACACTTGtctacatatatattcct
Cloning Inducible miRNA 2	I miR-s	gaTTTCACGAATACTTGAGGCTAtctctcttttgtattcc
	II miR-a	gaTAGCCTCAAGTATTCGTGAAAtcaaagagaatcaatga
	III miR*s	gaTAACCTCAAGTATACGTGAATtcacaggtcgtgatatg
	IV miR*a	gaATTCACGTATACTTGAGGTTAtctacatatatattcct
qPCR	F	AGCATTTTCCCAGGCACCACAAC
	R	TGCGACTTCTCCACCTCTCCAC
**NBR1 (AT4G24690)**		
qPCR	F	GAGGACCCAGACCGGAAGG
	R	GACAAACACGACGAGGATGC
**Reference genes**		
ACT2/8 (AT1G49240)	F	CCGAGCAGCATGAAGATTAAG
	R	CATACTCTGCCTTAGAGATCCACA
AT4G33380	F	GTGTTGTAACGGCTTGAGCA
	R	TGTTTGCATCTTTGGTACGG
