# Supplementary material for: Phosphatidylinositol-4-phosphate controls autophagosome formation in Arabidopsis thaliana
Source: Nat Commun. 2022 Jul 28;13:4385. doi: 10.1038/s41467-022-32109-2 (PMC9334301; doi:10.1038/s41467-022-32109-2)
Supplement: Supplementary file 3 — Description of Additional Supplementary Files [file 41467_2022_32109_MOESM3_ESM.pdf]

Description of Additional Supplementary Files

File Name: Supplementary Movie 1
Description: Time series of GFP-ATG8a roots in +N conditions for 30 minutes and then stained with propidium iodide. Movie shows little cytoplasmic movement; propidium iodide staining is observed in the apoplast. Time series were performed by acquiring 30 frames over 145s. See Supplementary Fig. 3 a,b for additional information. Scale bar: 10 µm.

File Name: Supplementary Movie 2
Description: Time series of GFP-ATG8a roots in -NC +DMSO for 30 minutes and then stained with propidium iodide. Movie shows cytoplasmic movement and an induction of GFP-ATG8a puncta compare to +N conditions (see Supplementary Movie 1); propidium iodide staining is observed in the apoplast. Time series were performed by acquiring 30 frames over 145s. See Supplementary Fig. 3 a,b for additional information. Scale bar: 10 µm.

File Name: Supplementary Movie 3
Description: Time series of GFP-ATG8a roots in -NC + 60µM PAO for 30 minutes and then stained with propidium iodide. Movie shows reduced cytoplasmic movement and GFP-ATG8a puncta compared to -NC +DMSO conditions (see Supplementary movie 2); propidium iodide staining is observed in the apoplast. Time series were performed by acquiring 30 frames over 145s. See Supplementary Fig. 3 a,b for additional information. Scale bar: 10 µm.
